# Plant-Based and Plant-Predominant Diets Among Healthcare Students: A Systematic Review

**DOI:** 10.7759/cureus.87294

**Published:** 2025-07-04

**Authors:** Raman Abbaspour, Sarah E Weisbrodt, Samal Nauhria, Madeline Chan, Ali Kasim Rawji, Parham Bokaei Jazi, Sabyasachi Maity

**Affiliations:** 1 Temerty Faculty of Medicine, University of Toronto, Toronto, CAN; 2 School of Podiatric Medicine, University of Texas Rio Grande Valley, Harlingen, USA; 3 Leadership Training, Civil Service College, Cayman Islands Government, George Town, CYM; 4 Joe R. and Teresa Lozano Long School of Medicine, University of Texas Health San Antonio, San Antonio, USA

**Keywords:** academic performance, cardiovascular and metabolic health, dietary adherence, medical and health sciences students, mental well-being, nutritional knowledge, plant-based diet, vegetarianism

## Abstract

This systematic review explores the effects of vegetarian diets on the physical and mental well-being of health sciences students, a demographic known for high academic and psychological stress. We conducted a comprehensive search across major databases, adhering to Preferred Reporting Items for Systematic Reviews and Meta-Analyses (PRISMA) guidelines, and identified 22 relevant studies. Our findings suggest that vegetarian diets are associated with lower incidences of chronic diseases such as coronary heart disease and obesity and promote better cardiovascular and metabolic health outcomes compared to non-vegetarian diets. Notably, vegetarian students could display lower systolic blood pressure and waist circumferences. Contrary to the positive physical health outcomes, the review presents mixed results concerning the mental health impacts, with some studies indicating no significant effects and others suggesting potential risks for increased anxiety and disordered eating behaviors. The review underscores the need for further research into the nuanced impacts of vegetarian diets on this specific population, suggesting that while the physical health benefits are evident, the psychological effects require deeper investigation to fully understand their scope and mechanisms.

## Introduction and background

A vegetarian diet is commonly described as a subtype of the plant-based diet in which all types of flesh are excluded [1]. Other commonly seen types of plant-based diets include the vegan diet, which also excludes milk and eggs, as well as the Mediterranean diet, which permits a small amount of animal foods [1]. Vegetarianism is widely practiced for a variety of personal and cultural beliefs, including the practice of religions that emphasize nonviolence and respect for all living beings [2]. Additionally, the recent discovery of potential health benefits linked to a vegetarian diet has contributed to an increase in its practice [3]. Worldwide, vegetarian diet adherence is greatest in Asia, followed by Africa and the Middle East. The lowest prevalence of a vegetarian diet is seen in North America alongside Europe [2].

The impact of a vegetarian diet on the general population has been described as largely positive, with many studies demonstrating an optimistic impact on health outcomes when compared to alternative diets [4]. A vegetarian diet has been associated with reduced incidence of many conditions, including type 2 diabetes, hypercholesterolemia, and overweight [5]. The risk of coronary heart disease (CHD) is decreased by 40% in vegetarians, and plant-based diets have even shown the ability to reverse CHD progression [6]. Vegetarian diets are linked to lower concentrations of blood total cholesterol and high-density lipoprotein (HDL) cholesterol when compared with nonvegetarian diets, as well as decreased risk of multiple cancers [7]. Furthermore, individuals following plant-based diets are more likely to consume a greater quantity of vital nutritional components such as potassium, magnesium, folic acid, fiber, and vitamins C and E while also weighing less than their non-vegetarian counterparts [8]. While plant-based diets have been associated with positive effects on mental wellbeing for some individuals, the literature also notes mixed findings and highlights potential mental health concerns for others [8].

When looking at the impact of the vegetarian diet on student populations, research supports an improvement in physical health, though we see mixed results regarding its impact on students’ mental health. In one study, vegetarian diet was not considered to be linked to positive or negative mental health in German university students, while in Chinese university students, the diet predicted a slight increase in anxiety and depression over time [9]. However, vegetarian university students may be at heightened risk for disordered eating behaviors, especially when the diet is pursued for health or weight-loss purposes [10]. Regarding physical health, multiple studies indicate that vegetarian university students have a lower body mass index (BMI) than their peers, which is associated with a lower risk of cardiovascular disease and a decrease in instances of obesity [11,12]. A study by Olfert et al. suggests that while there are no clear differences in physical health indicators between vegetarians and nonvegetarians, vegetarian university students have significantly lower systolic blood pressure and waist circumferences than their nonvegetarian peers [13]. In terms of academic performance, a nonvegetarian diet has a negative impact on student mood, potentially impairing learning and academic success [14].

The primary objective of this systematic review is to explore the impact of vegetarian diets among health sciences students, a population known to experience high levels of academic and psychological stress [10]. These students endure rigorous academic schedules, clinical placements, and high-stakes examinations that contribute to their elevated stress levels. Given that dietary choices play a significant role in both physical and mental well-being, understanding the potential benefits and prevalence of vegetarian diets within this cohort is essential. We are interested in seeing if adopting a vegetarian diet may serve as a cost-effective and practical approach to alleviating student stress during these demanding academic years, much like other holistic practices such as yoga [15].

## Review

Methods

This systematic review was conducted in accordance with the Preferred Reporting Items for Systematic Reviews and Meta-Analyses (PRISMA) guidelines [16].

Eligibility Criteria

The inclusion criteria for the studies focused on participants who were medical, dental, nursing, or other healthcare/allied health students and involved interventions related to vegetarian, vegan, or plant-based diets. The exclusion criteria ruled out studies involving undergraduate, graduate, or school students not enrolled in medical or allied health programs and any interventions that included meat-based diets. Additionally, non-peer-reviewed materials like editorials, letters, commentaries, reviews, conference posters, preprints, and dissertations were excluded from the review.

Information Sources

A comprehensive search was performed in electronic databases including PubMed (US National Library of Medicine, National Institutes of Health), Scopus, Embase, and Google Scholar. We searched for studies published in English from the inception of the databases up to December 2024.

Search Strategy

The search terms used were related to dietary habits, such as “vegetarian”, “plant-based diet”, “Mediterranean diet”, and “healthcare students”, encompassing medical, dental, nursing, and other allied health professions students.

Selection Process

Five reviewers (RA, AR, MC, SW, PJ) independently screened the titles and abstracts of retrieved articles using the Covidence systematic review software [17]. Studies were selected for full review if they met the following inclusion criteria: studies that reported on the effects of plant-based diet on healthcare/allied health students conducted in any geographic location. We considered all types of observational and experimental study designs, including cross-sectional, cohort, and randomized controlled trials. Disagreements were resolved through discussion or, if necessary, consultation with a sixth reviewer (SM).

Data Collection Process

Data extraction was conducted by five reviewers (RA, AR, MC, SW, PJ) using a standardized Excel spreadsheet (Microsoft Corporation, Redmond, Washington, United States) to capture relevant study details. Extracted information included authors, year of publication, geographical location, study duration, participant demographics (e.g., age range, professional background), sample size, type of dietary intervention, and the outcomes measured relating to stress, cognitive performance, physiological changes, and knowledge of vegetarian diet. We also made note of the conclusion of the articles as well as their limitations. Any discrepancies were resolved by consensus, involving the sixth reviewer (SM) when necessary.

Risk of Bias

Risk of Bias (RoB) assessment was conducted using the JBI Critical Appraisal Tool [18] to evaluate the methodological quality of the included studies. The assessment focused on key domains such as selection bias, confounding, and reporting reliability.

Results

**Figure 1:**
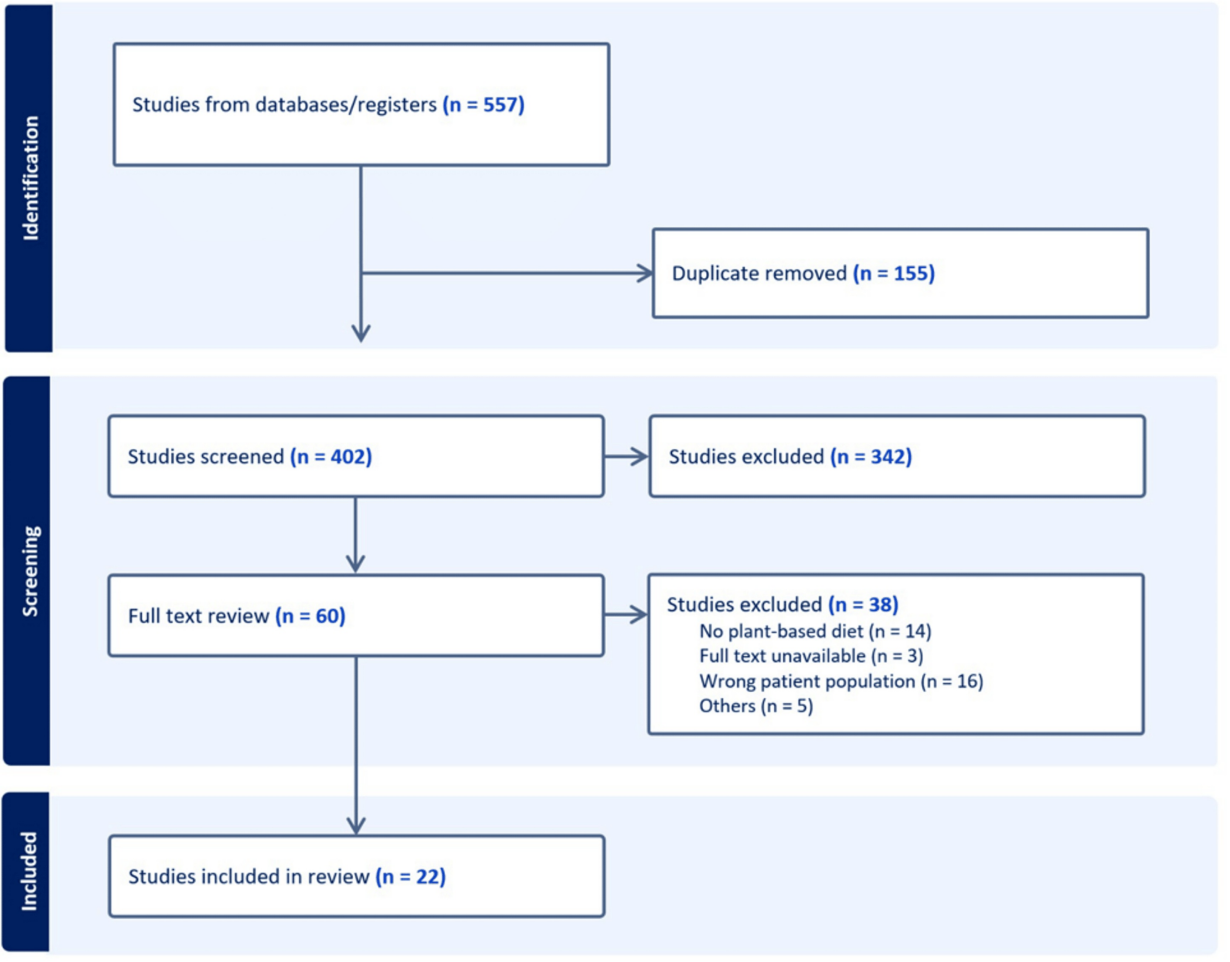
PRISMA flow diagram summarizing the study selection process. A total of 557 records were identified from databases and registers. After removing one duplicate, 402 studies were screened based on titles and abstracts, resulting in the exclusion of 342 studies that did not meet the eligibility criteria. Sixty studies underwent full-text review, of which 38 were excluded for reasons including no plant-based diet intervention (n = 14), unavailable full text (n = 3), wrong patient population (n = 16), and other reasons (n = 5). Ultimately, 22 studies were included in the systematic review. PRISMA: Preferred Reporting Items for Systematic Reviews and Meta-Analyses

Our systematic review process commenced with an initial search that yielded a total of 557 articles (402 after duplicate removal). This number encompassed results from database searches and hand-searching methods. After a preliminary screening of titles and abstracts, 342 articles were excluded based on criteria such as irrelevance to the study objectives or being conducted outside the scope of healthcare student populations. Upon a detailed full-text review, an additional 38 articles were excluded due to reasons such as having the wrong patient population, not focusing on a plant-based diet, unavailability of full-text, or non-original research designs. Ultimately, 22 articles met our stringent inclusion criteria and were included in the systematic review. The flow of study selection is illustrated in Figure 1, represented by a PRISMA flowchart. Table 1 also provides a summary of these articles.

**Table 1:** Overview of included studies on plant-based diet interventions among healthcare students A comprehensive synthesis of the 22 included studies, detailing each study’s authors, year of publication, research type, geographic location, participant demographics, and intervention type which includes the diet assessed and/or the educational program delivered. The conclusions drawn by each study are also summarized to provide a clear overview of the existing evidence on the effects of plant-based diets among healthcare students. WFPB: whole-food, plant-based; SBP: systolic blood pressure; DBP: diastolic blood pressure; IBS: irritable bowel syndrome: PREDIMED: PREvención con DIeta MEDiterránea: PA: physician assistant

Author and year	Research type	Location and population	Type of diet/program	Conclusion
Choudhary et al., 2016 [19]	Experimental study	India: 100 healthy female medical students, 19-25 years	Group I: Vegetarians (n=45); Group II: Eggetarians (n=25); Group III: Nonvegetarians (n=30)	- Nonvegetarians showed greater alterations in cognitive and physiological parameters, likely linked to higher BMI. - Long-term vegetarian diets might enhance vagal heart regulation without boosting sympathetic activity.
Morton et al., 2022 [20]	Cross-sectional survey	United States: 182 medical students; age unspecified	WFPB diet or variation: current followers (n=28), past followers (n=52)	- 22% agreed they received sufficient training, 41% supported a focus on WFPB diets in medical curricula. - WFPB followers were significantly more likely to recommend it to patients and viewed it as a feasible and beneficial approach to managing chronic diseases.
Fernández-Medina et al., 2020 [21]	Cross-sectional survey	Spain: 334 nursing students; mean age 21.84 years	Mediterranean: good adherence (n=35), poor adherence (n=299)	Good sleep quality and adherence to the Mediterranean diet improve academic performance in nursing students
Navarro-González et al., 2014 [22]	Cross-sectional survey	Spain: 318 nursing students; mean age 20 years	Mediterranean: good adherence (n=138), poor adherence (n=10)	- Mediterranean diet adherence is associated with BMI - Adherence was greater in 4^th^ year students compared to 1^st^ years. - Better adherence in 4^th^ years who live away from home than 4^th^ years who live at home
González-Sosa et al., 2023 [23]	Cross-sectional survey	Spain: 589 medical students; mean age 22 years	Mediterranean: good adherence (n=347), low adherence (n=242)	- Future physicians, compared to the overall university population, probably show greater concern for their health OR have more nutrition knowledge - Adherence increases with age - It remains unclear whether moderate alcohol belongs in a balanced, healthy diet
Ibáñez-Del Valle et al., 2023 [24]	cross-sectional survey	Spain: 289 nursing students or podiatry; mean age 20.60 years	Mediterranean diet: 35.6% of the students showed good adherence	- Adherence to the Mediterranean diet is inversely associated with depressive symptoms among nursing students. - The study’s findings support implementing prevention and health promotion programs in universities.
Samudhrasri et al., 2021 [25]	Case-control observational	India: 20 dentistry students; age range 17-20 years	Group 1: Vegetarians Group, 2: Non-vegetarians	Vegetarians had significantly: - Decreased SBP/DBP, BMI - Increased heart rate
Ring et al., 2019 [26]	Surveys, Qualitative Feedback, Observational Data	United States: Medical students (Cohort 1: 9, Cohort 2: 12); Mean age ~22 years	Plant-based culinary medicine elective combining lectures, hands-on cooking, and service learning.	Such programs are feasible and effective for improving nutrition counselling confidence and dietary behaviours.
Cherpak-Castagna et al., 2022 [27]	Cross-sectional survey	United States: 208 clinical nutrition students	Various diets including vegan/vegetarian and whole foods.	- Vegan/vegetarian diets are among the most common diets followed by clinical nutrition students - The most reported reasons for following a diet were health optimization and food allergy/intolerance/sensitivity.
Banerjee et al., 2024 [28]	Cross-sectional survey	India: 397 medical students; age distribution unspecified	114 vegetarian, 110 mostly vegetarian, 173 non-vegetarian	No statistically significant association between dietary habits and the occurrence of IBS
Suwalska et al., 2022 [29]	Cross-sectional study	Poland: 227 students; mean age 21 years	Mediterranean diet	Highlights the need for mental health and nutritional interventions for university students. Diet quality is linked to mental health outcomes.
Singh et al., 2016 [30]	Cross-sectional study	India: 162 medical students, staff members and members of their families; ages 7-75 years	Comparison of vegetarian and omnivorous diet	- Diet, gender, and smoking influence lipid profiles. - Results highlight the potential for non-pharmacological interventions to improve metabolic health.
Huang et al., 2024 [31]	Observational study	United States: 46 medical students; ages not specified	WFPB diet, nutrition seminars, health screenings, and self-paced dietary change	- Short-term WFPB diet reduced weight and blood pressure. - Nutrition seminars effectively increased understanding and motivation among students to integrate nutrition education into medical practice
Baydemir et al., 2018 [32]	Cross-sectional study	Turkey: 354 medical students; mean age ~20 yrs	Mediterranean diet	- Medical students demonstrated low adherence to the Mediterranean diet. - Female students had higher adherence than males. - Better adherence was linked to non-smoking, lower BMI, and regular exercise
Flynn et al., 2019 [33]	Interventional Study with Questionnaire	United States: 44 medical students; 23.9 ± 2.2 years	Plant-based diet, Cooking program	- A 4-week cooking program can improve diet and nutrition knowledge, and increase plant-based diet in medical students.
Kommana et al., 2015 [34]	Prospective Imaging Pilot Study	United States: 37 healthy South Asian medical students; mean age: 23 ± 1 yrs	Comparison of dietary lifestyles (vegetarian vs. nonvegetarian)	- Vegetarians had lower autofluorescence levels, suggesting reduced oxidative stress in the retina - A well-balanced vegetarian diet may promote long-term retinal health
Sanne and Bjørke-Monsen, 2022 [35]	cross-sectional study	Norway: 394 medical students; 18–47 years	Former or current vegetarians (24%) versus always omnivores (76%)	- Nutritional knowledge regarding vegetarian and vegan diets was not satisfactory among Norwegian medical students and did not differ according to the former and current diet.
Najafi et al., 2018 [36]	nested case-control study	Iran: 100 health science students from 7 faculties	Dysmenorrhea: n= 46, Age: 21.89 ± 1.43, Control: n=54, Age: 21.92 ± 1.83	A high consumption of sugars, salty snacks, sweets and desserts, tea and coffee, salt, fruit juices and added fat, is associated with an increased risk of dysmenorrhea among young women.
Spencer et al., 2007 [37]	Longitudinal survey	United States: 1849 medical students	Vegetarian: n=150 at freshman (8.1%), n=105 at junior (7%), n=51 at senior (6%)	- Vegetarianism prevalence is higher among med students than among other adults. - Prevalence declined during medical school. - No association between vegetarianism among medical students and their nutrition counselling practices.
Gianfredi et al., 2018 [38]	Cross-sectional study	Italy: 117 nursing students; age 23.7 ± 4.8 (20-43)	Mediterranean Diet (measured by PREDIMED score)	- Statistical significant association between PREDIMED score and BMI, smoking habit, quality of life, sleep disturbances, and academic progress.
Hanna et al., 2021 [39]	Cross-sectional study	Ireland: 101 MPharm students; 21 or 22 yrs	Veganism: Yes/Maybe (n=17) vs No/Never (n=75)	- Significant gap in pharmacy students’ education about veganism. - Students show a lack of confidence in discussing vegan-related topics.
Charles et al., 2023 [40]	Longitudinal survey	United States: 80 PA students; mean age 31.4	Plant-Based Nutrition educational session	The virtual nutrition education and a culinary medicine curriculum increased nutrition knowledge, confidence in nutrition skills, and perceived importance of clinical nutrition

The results of the RoB evaluation are visualized in Table 2. However, two observational studies, by Ring et al. [36] and Huang et al. [31], employed different assessment criteria and are therefore not included in Table 2. According to the JBI tool, Ring et al. scored 9/10, indicating a low risk of bias, while Huang et al. received a 7/10, suggesting a moderate risk of bias.

**Table 2:** Risk of Bias assessment using the JBI Critical Appraisal Tool. The table illustrates the methodological quality of the included studies across key domains. Y indicates a low risk of bias, U represents an indeterminate risk, and N signifies a high risk of bias. Two observational studies (Ring et al. [26] and Huang et al. [31]) were assessed using different criteria and are not included in this table.

Author and Year	Q1	Q2	Q3	Q4	Q5	Q6	Q7	Q8	Q9	Overall Score	Include
Choudhary et al., 2016 [19]	U	Y	Y	Y	U	N	Y	Y	Y	6	Y
Morton et al., 2022 [20]	U	U	Y	U	N	N	Y	U	Y	3	Y
Fernández-Medina et al., 2020 [21]	Y	N	Y	Y	Y	N	Y	Y	Y	7	Y
Navarro-González et al., 2014 [22]	Y	N	Y	Y	N	Y	Y	Y	Y	7	Y
González-Sosa et al., 2023 [23]	Y	Y	Y	Y	U	Y	Y	Y	Y	8	Y
Ibáñez-Del Valle et al., 2023 [24]	U	Y	Y	U	N	N	Y	N	Y	4	Y
Samudhrasri et al., 2021 [25]	N	Y	Y	U	Y	Y	Y	N	Y	6	Y
Cherpak-Castagna et al., 2022 [27]	U	U	Y	U	Y	Y	Y	N	Y	5	Y
Banerjee et al., 2024 [28]	N	Y	U	U	N	N	Y	U	Y	3	Y
Suwalska et al., 2022 [29]	N	Y	Y	Y	N	N	Y	Y	Y	6	Y
Singh et al., 2016 [30]	N	Y	Y	U	Y	N	Y	Y	Y	6	Y
Baydemir et al., 2018 [32]	U	Y	Y	U	N	N	Y	Y	Y	5	Y
Sanne and Bjørke-Monsen, 2022 [35]	N	U	N	N	Y	N	N	N	N	1	Y
Gianfredi et al., 2018 [38]	N	N	N	N	Y	N	N	U	N	1	Y
Hanna et al., 2021 [39]	U	U	Y	U	Y	N	U	Y	Y	4	Y
Flynn et al., 2019 [33]	U	U	Y	U	N	N	Y	Y	Y	4	Y
Najafi et al., 2018 [36]	N	Y	Y	Y	N	N	Y	U	N	4	Y
Spencer et al., 2007 [37]	N	Y	Y	Y	Y	N	Y	Y	Y	7	Y
Charles et al., 2023 [40]	N	Y	Y	Y	Y	N	Y	Y	Y	7	Y
Kommana et al., 2015 [34]	Y	Y	Y	Y	N	N	Y	Y	Y	7	Y

Physiological Effects

In synthesizing the results pertaining to the physiological effects of plant-based diets among healthcare student populations, several noteworthy findings emerged from the analyzed studies. Table 3 summarizes the findings.

**Table 3:** Summary of the physiological effects of plant-based diets among healthcare student populations, as reported in 14 of the 22 included studies. The findings include associations between dietary patterns (vegetarian or Mediterranean) and physiological outcomes such as heart rate variability, BMI, blood pressure, lipid profiles, menstrual health, and exercise habits. Significant associations were observed for improved BMI, blood pressure, and exercise patterns with adherence to plant-based or Mediterranean diets, while mixed results were noted for lipid parameters and menstrual health. This table provides an overview of the physiological impacts of plant-based dietary practices in healthcare students. SBP: systolic blood pressure; DBP: diastolic blood pressure; HR: heart rate; IBS: irritable bowel syndrome; T-C: total cholesterol; PL: phospholipids; TL: lotal lipids; MD: Mediterranean diet

Name and author	Results
Choudhary et al., 2016 [19]	- Non-vegetarians showed a more significant alteration of heart rate variability (HRV) during the luteal phase of the menstrual cycle (2040 ± 295 Veg vs 1178 ± 137 non-Veg for total spectral power)
Fernández-Medina et al., 2020 [21]	- Mediterranean diet adherence is associated with better sleep
Navarro-González et al., 2014 [22]	- Students with higher BMI (>25) had a slightly higher adherence to the MD (KIDMED score of 7.3 vs. 7.0 for BMI < 25).
González-Sosa et al., 2023 [23]	- MD adherence was significantly associated with frequency (P < 0·001), intensity (P = 0·003), and volume (P = 0·001) of exercise
Samudhrasri et al., 2021 [25]	Vegetarians had significantly decreased SBP (-11.00 mmHg)/DBP(-10.80 mmHg), BMI (-4.84), increased HR (14.90 bpm)
Banerjee et al., 2024 [28]	No significant association between vegetarianism and IBS.
Suwalska et al., 2022 [29]	Greater adherence to MD linked with lower BMI (an association was observed between high scores of specific eating behaviors and body weight, adherence to the MD, and consumption of specific product groups (sweets, alcohol) ) and better overall diet quality (many factors)
Singh et al., 2016 [30]	- Vegetarians had lower midline estimating statistic of rhythm (MESOR) of T-C, PL, and TL
Huang et al., 2024 [31]	- Decrease in weight (-0.9 (-2.2, .0, P < .0461) pounds) - BP (differences in systolic and diastolic BP were-5.0 (-9.0, -0.5, P < .049) and -7.0 (-11.0, -2.0, P < .0037) mmHg, respectively.) - Non significant improvement in lipid parameters
Baydemir et al., 2018 [32]	BMI was higher among students with lower adherence scores (22.2 ± 3.2, 21.6 ± 2.9, and 21.3 ± 3.5 kg/m2 for the poor, average, and good adherence)
Kommana et al., 2015 [34]	- Vegetarians had statistically significant lower levels of lipofuscin in the papillo-macular zone compared to nonvegetarians (75.49 ± 18.72 vs 90.71 ± 16.17)
Sanne and Bjørke-Monsen, 2022 [35]	- No difference in BMI (Vegetarian: 22.3 [20.4, 23.5], Omnivore: 22.5 [20.8, 24.2], P = 0.13). - No difference in regular exercise (Vegetarian: 88%, Omnivore: 92%, P = 0.29). - No difference in regular alcohol consumption (Veg:(81%) vs Omnivore: (83%) 0.73*). - No difference in Regular use of tobacco (Cigarettes: Veg: (2%) 2 (1%) 0.22 and Snuff Veg: (11%) (11%) 0.85)
Najafi et al., 2018 [36]	- No association between “lacto-vegetarian” and risk of dysmenorrhea.
Gianfredi et al., 2018 [38]	- Better adherence to MD is correlated with lower smoking rates (p-value = 0.0127) - Better adherence associated with a more favorable BMI (p-value = 0.0127) - Higher adherence to the MD is associated with a better quality of life among the students (p-value = 0.0480)

Several studies highlighted the positive impact of plant-based and Mediterranean diets on cardiovascular health markers. For instance, following a plant-based diet showed a significant reduction in both systolic and diastolic blood pressure among vegetarians, along with decreased BMI and an increase in heart rate [25,31]. Similarly, Choudhary et al. reported that nonvegetarians experienced more significant alterations in heart rate variability (HRV) during the luteal phase of the menstrual cycle, suggesting potential cardiovascular stability in vegetarians [20]. Furthermore, Singh et al. found that vegetarians had lower midline estimating statistic of rhythm (MESORs) of total cholesterol, phospholipids, and triglycerides, indicating better lipid profiles [29]. This is while Huang et al. noted non-significant improvements in lipid parameters [31]. These studies collectively suggest that diets rich in plant-based components can lead to notable improvements in cardiovascular health among healthcare students.

In addition to cardiovascular outcomes, changes in weight and BMI were also observed in following a plant-based diet [22,23,25]. In terms of adherence, Suwalska et al. reported that greater adherence to a Mediterranean diet was linked with lower BMI and improved overall diet quality [29]. Similarly, Baydemir et al. found that BMI was higher among students with lower adherence to vegetarian diets, indicating that the level of adherence might influence the effectiveness of the diet on weight management [32]. Gianfredi et al. also supported this, noting that better adherence to a Mediterranean diet correlated with more favorable BMI outcomes [38]. On the contrary, Sanne and Bjørke-Monsen did not report a significant difference between vegetarian and omnivore students in BMI [35].

The results also showed that following a plant-based diet can improve lifestyle. For example, it was shown that the Mediterranean diet can enhance sleep quality [21,38]. Additionally, the Mediterranean diet is associated with lower smoking rate [38] and frequency, intensity, and volume of exercise [23].

Psychological Effects

Out of the 22 studies reviewed (Table 1) on the effects of vegetarianism and related dietary habits, only five [19,21,24,29,38] focused on psychological or academic components relevant to medical and nursing students. These studies provide valuable insights into how dietary patterns influence mental health and academic performance.

One study found that nonvegetarians experienced a significant prolongation of visual and auditory reaction times during the luteal phase of menstruation, indicating a potential connection between dietary habits and sensory-motor performance, through hormonal fluctuations [19]. Additionally, adherence to the Mediterranean diet was strongly linked to improved self-efficacy and academic performance, underscoring the educational benefits of healthy dietary practices [21]. This relationship was further corroborated by evidence showing a statistically significant association between Mediterranean diet adherence and academic progress [38].

Psychological effects were also prominent in these studies. Poor adherence to the Mediterranean diet was significantly associated with increased depressive symptoms, indicating the importance of dietary choices for mental health [24]. This finding aligns with another study where higher cognitive restraint, a factor linked to better mental well-being, was associated with Mediterranean diet adherence [29].

These five studies highlight the critical psychological and academic dimensions of dietary habits among medical and nursing students, suggesting that mindful adherence to balanced diets, such as the Mediterranean diet, can positively influence mental health and academic outcomes.

Knowledge and Perception

A review of studies on medical students’ knowledge and perceptions regarding vegetarianism and plant-based diets reveals key trends in awareness, attitudes, and confidence (Table 4). These insights demonstrate how dietary knowledge influences students’ perceptions and their readiness to incorporate nutrition into healthcare practice.

**Table 4:** Overview of knowledge and perceptions regarding plant-based diets, as reported in 11 of the 22 included studies. The table summarizes key information, including the authors, publication year, and the main findings related to students’ awareness, understanding, and attitudes toward plant-based diets. Topics covered include knowledge of dietary benefits, confidence in recommending plant-based diets, self-perception of health, and familiarity with diet-related risks (e.g., nutrient deficiencies). The table provides a detailed synthesis of the studies addressing educational and perceptual aspects of plant-based diets among healthcare students. WFPB: whole-food, plant-based

Author and Year	Results
Morton et al., 2022 [20]	Current followers compared to non-followers: - Significantly more likely to recommend a WFPB diet to patients across all proposed reasons and in general (59% vs 17%, P < .001) - The past followers group was also significantly more likely to recommend a WFPB diet to patients in general than those who were not past followers (80% vs 46%, P < .001).
Ibáñez-Del Valle et al., 2023 [24]	- Participants with better adherence to MD reported a better self-perception of health. (Not significant but trend toward greater adherence among those with a better self-perception of their personal health, p = 0.067)
Ring et al., 2019 [26]	Improved knowledge and confidence in plant-based diets (e.g., preparation, substitution).
Cherpak-Castagna et al., 2022 [27]	- 13% follow vegetarian/vegan diet - students following a veg/vegan diet were more likely to recommend it
Huang et al., 2024 [31]	Students gained a significantly better understanding of the link between diet and health and were more likely to advocate for integrating nutrition into the medical curriculum
Baydemir et al., 2018 [32]	First- and third-year medical students displayed low adherence to the Mediterranean diet, despite their knowledge of healthy living (The mean KIDMED score of the female and male students was 4.0 ± 1.9 and 3.6 ± 2.0 points (p = 0.037) + male versus female students: poor score (36.9% vs. 49.7%, respectively) average score (47.8%, vs. 61.0% vs. respectively) good score (2.5%, vs. 2.1% respectively)
Flynn et al., 2019 [33]	- Significant improvement in nutrition knowledge (baseline 43.4 + 18.9 vs. FU 83.0 + 16.1; p = 0.000) - Increased awareness and use of plant-based diets (At baseline, plant-based/vegetarian main meals/week were 3.2 + 2.4 vs. FU 4.2 + 2.1 (p = 0.005) + more factors such as less red meat and using the recipe)
Sanne and Bjørke-Monsen, 2022 [35]	- More vegetarian students than omnivore students considered a vegetarian diet to be healthier than an omnivore diet (44 vs 13)
Spencer et al., 2007 [37]	- The most commonly cited reason for self-reported vegetarianism was health (66%). - vegetarianism was not associated with counseling patients on nutrition
Hanna et al., 2021 [39]	- Students who were vegan or would consider becoming vegan had similar knowledge scores to the omnivore group - only around one in three felt confident discussing veganism with patients or other healthcare professionals
Charles et al., 2023 [40]	- Knowledge scores increased by 30%. - Greater confidence in dietary history-taking (55% vs. 95%; p = 0.001) and nutritional counseling (53% vs. 84%; p = 0.003). - More agreement that dietary interventions can reverse chronic disease. (type 2 diabetes (74% vs. 97%; p = 0.027) and coronary artery disease (66% vs. 92%; p = 0.039)) - No change in attitudes toward nutrition’s role in care, personal responsibility, patient behavior, or primary care’s role.

Medical students currently following whole-food, plant-based (WFPB) diets were significantly more likely to recommend such diets to patients, citing their familiarity with the diet’s feasibility and health benefits. Interestingly, past followers were also more likely than non-followers to advocate for WFPB diets, though to a lesser extent than current adherents [20]. Similarly, students adhering to the Mediterranean diet reported a stronger self-perception of health, reinforcing the personal benefits of healthy dietary practices [25]. Furthermore, students adhering to vegetarian or vegan diets were not only more likely to recommend these diets but also demonstrated a better understanding of their health benefits [27].

Participation in dietary education programs significantly improved students’ knowledge and confidence in plant-based diets, including practical aspects such as meal preparation and ingredient substitution [26]. This is particularly relevant for medical students, as enhanced dietary knowledge can influence their future interactions with patients. Nutrition-focused seminars also strengthened students’ understanding of the diet-health connection, with many participants advocating for the integration of nutrition into medical curricula. These programs often led to improved mindfulness about eating [31]. However, despite awareness of healthy living principles, first- and third-year medical students demonstrated low adherence to the Mediterranean diet, highlighting a persistent gap between knowledge and practice [32].

Studies also reveal differences in dietary knowledge among students based on their dietary habits. Vegetarian students were more likely than omnivorous students to perceive vegetarian and vegan diets as healthier and to recognize risks such as vitamin B12 deficiency. In contrast, omnivorous students displayed greater awareness of other micronutrient deficiencies [35]. Importantly, targeted nutrition education significantly increased knowledge scores, boosting students’ confidence in dietary history-taking and nutritional counseling. However, attitudes toward the importance of nutrition in chronic disease care and changes in personal dietary habits showed limited improvement [40]. Consistent with this, Flynn et al. demonstrated that even a short, four-week program could enhance diet and nutrition knowledge while promoting plant-based dietary practices among medical students [33].

Nevertheless, some studies found no strong association between students’ diets and their probability of counselling patients on vegetarianism, as personal health considerations appeared to be the primary motivator behind vegetarian dietary choices [37,39]. Notably, gaps in knowledge persist, with only one in three students reporting confidence in counseling about veganism [39].

Impact of Lifestyle

Emerging evidence underscores the complex interplay of academic year, gender, age, and lifestyle factors on dietary adherence among medical and nursing students. These findings reveal significant trends that can inform interventions aimed at enhancing dietary practices and addressing gaps between knowledge and implementation.

Advanced-year students consistently demonstrate greater adherence to the Mediterranean diet compared to their earlier-year counterparts. For instance, fourth-year nursing students in Spain exhibited higher adherence than first-year students, with those living away from home showing even greater adherence [22]. Similarly, medical students in their final three years demonstrated significantly better adherence to the Mediterranean diet than those in their initial years [23]. Age also plays a critical role; older nursing students with good Mediterranean diet adherence were more likely to maintain the diet [24]. Despite these insights, adherence to the Mediterranean diet remains low in certain contexts. For instance, medical students at Kocaeli University reported low adherence despite their awareness of healthy living principles [32]. This persistent gap between knowledge and practice emphasizes the need for targeted interventions designed to bridge this divide.

Dietary education programs have demonstrated a measurable impact on students’ dietary habits and understanding. For example, a four-week plant-based cooking program significantly increased medical students’ adherence to plant-based diets [33]. Other similar programs have promoted increased fruit and vegetable intake while reducing meat consumption [26]. Furthermore, students with higher adherence to healthy diets displayed improved understanding and integration of healthy behaviors into their lifestyles [29].

Lifestyle factors also exert a considerable influence on dietary adherence. Certain habits, such as smoking, were associated with significantly lower adherence to the Mediterranean diet [24]. Conversely, students who consumed alcohol at least twice monthly reported higher Mediterranean diet adherence, indicating a complex and potentially cultural interaction between lifestyle behaviors and dietary patterns [23].

Gender disparities further highlight differences in dietary adherence. Female students consistently showed better adherence to the Mediterranean diet compared to their male counterparts, as observed at Kocaeli University, Turkey [32]. This aligns with broader research indicating that women generally exhibit more health-conscious behaviors than men.

Vegetarianism among students revealed distinct personal and professional associations. Vegetarian students were more likely to be female, adhere to specific religious and political beliefs such as Buddhism or Hinduism, and value nutrition counseling in their future practice [37]. They also displayed greater awareness of nutrients critical for health, such as those supporting retinal function [34]. However, studies conducted in Norway found no significant differences between vegetarians and omnivorous students in alcohol or tobacco use, further highlighting the nuanced relationships between lifestyle and dietary choices [35].

Discussion

Physiological Effect

Cardiovascular health: Our review found that vegetarianism is associated with better cardiovascular health. Previous literature suggests that this association arises because a vegetarian diet improves the gut microbiome and provides superior nutrient sources.

Dysbiosis, or gut microbiome imbalance, disrupts the immune system, causing inflammation that contributes to poor cardiovascular health [41]. Dysbiosis has been linked to adverse cardiovascular outcomes (e.g., atherosclerotic cardiovascular disease, heart failure) and associated risk factors (e.g., hypertension, diabetes mellitus, chronic kidney disease, obesity). Vegetarians have lower levels of pro-inflammatory bacteria, such as *Bacteroides*, *Ruminococcus*, and *Clostridia*, which produce trimethylamine N-oxide (TMAO). TMAO is associated with increased risks of cardiovascular disease, atherosclerotic plaque formation, heart attack, stroke, obesity, diabetes, and chronic kidney disease.

Conversely, vegetarians have higher levels of anti-inflammatory bacteria, such as *Prevotella*, *Faecalibacterium prausnitzii*, and *Xylanibacter* [41]. These bacteria produce short-chain fatty acids that improve cardiovascular risk factors by regulating blood pressure (e.g., renin secretion) and enhancing metabolism (e.g., satiety, insulin sensitivity, and glucose tolerance). A vegetarian diet enhances gut microbial diversity, stability, and resilience, which is key for cardiovascular health.

Secondly, a vegetarian diet provides nutrient sources that benefit cardiovascular health [41]. It is rich in polyphenols, which help prevent inflammation, oxidative DNA damage, and lipid peroxidation, key contributors to atherosclerosis. It also contains high levels of magnesium and potassium, which improve insulin sensitivity and reduce blood pressure, stroke risk, and cardiovascular disease. Meanwhile, a vegetarian diet typically contains lower amounts of sodium, high-density lipoprotein (HDL) cholesterol, and foods with a high glycemic index and load, all of which are risk factors for cardiovascular disease.

Body weight: Our review accords with previous knowledge that vegetarians typically have lower body weight or BMI, which contributes to their improved cardiovascular health [41]. This can be attributed to the reduced nutritional bioavailability of a vegetarian diet. Plant-based foods are high in fiber, cellulose, and large food particles that are not easily absorbed in the intestine. Instead, these nutrients are metabolized by colon microbiota, which increases the host’s resting metabolism. Fiber promotes satiety due to its lower energy density, providing fewer calories for a larger volume of food, and because it requires more chewing, causes stomach distension, and slows the transit time of food through the digestive system. These factors contribute to improved insulin sensitivity. The combination of reduced nutritional bioavailability and the satiation effects of fiber largely explains why vegetarians tend to have lower body weight.

Sleep: Two of our included studies found that a plant-based diet is linked to improved sleep quality [21,38]. Based on past literature, we theorize that this is due to its nutrient profile, anti-inflammatory effects, and positive impact on the microbiome [42]. Firstly, plant-based diets are rich in nutrients beneficial for sleep. They provide high levels of tryptophan, which is converted to serotonin and melatonin hormones that regulate the sleep-wake cycle. Legumes, high in vitamin B6, enhance this conversion, while vegetarian diets, rich in magnesium, lower cortisol and activate γ-aminobutyric acid (GABA) receptors, promoting central nervous system relaxation. Soybeans and legumes also contain isoflavones, which mimic estrogen and regulate sleep duration, quality, and efficiency. Leafy greens provide calcium, and insufficient intake is linked to poor sleep [42]. Secondly, inflammatory cytokines are associated with sleep disturbances. Plant-based diets, high in nitrates, promote nitric oxide production, which supports endothelial function and reduces inflammation. They also help prevent insulin resistance and lower insulin levels, both of which contribute to inflammation [42]. Finally, dysbiosis is linked to poor sleep. A plant-based diet enhances gut microbiome diversity and lowers the *Firmicutes*-to-*Bacteroidetes* ratio, leading to improved sleep quality, efficiency, and duration [42].

Psychological Effect

Our findings align with previous studies showing that a vegetarian diet is associated with improved cognitive function, enhanced academic performance, and a reduced risk of depression [43-45].

Vegetarian diets provide key nutrients associated with cognitive health, such as vitamin C and calcium, which function as neurotransmitters [46-48]. They are also rich in antioxidants, omega-3 fatty acids, and B vitamins, all of which are essential for neuronal function, synaptic plasticity, and neuroprotection [49]. As reducing inflammation improves cognition and reduces the risk of cognitive decline and neurodegenerative diseases, the anti-inflammatory properties of a vegetarian diet may further support cognitive health [50-52].

The nutrients in a vegetarian diet also support mood regulation to reduce the risk of depression. Fruits and vegetables contain folate, magnesium, vitamin C, and polyphenols, which enhance the signaling pathways of serotonin and dopamine, two neurotransmitters essential for mood regulation and commonly implicated in depression [53]. Vegetarian diets also provide amino acids, including lysine, tyrosine, methionine, tryptophan, arginine, and β-alanine, which all serve as precursors for the synthesis of dopamine and serotonin [54,55].

One possible explanation for the association between a vegetarian diet and academic success may lie in the healthier lifestyle often adopted by vegetarians, rather than the diet itself. Vegetarians typically engage in more physical activity and exhibit better overall health, which we believe may translate into greater self-efficacy due to their commitment to a healthier lifestyle [45,56,57]. Supporting this, Jochem observed that vegetarians report higher self-confidence with academic material compared to non-vegetarians, despite no significant differences in GPA [45]. Many vegetarians are also motivated by animal welfare and environmental sustainability [41,58,59]. They experience enhanced well-being through the altruistic purpose their lifestyle provides, which fosters a sense of self-efficacy. Thus, the psychological benefits of adhering to a vegetarian diet, such as a drive for self-improvement and a commitment to an altruistic lifestyle, may enhance self-efficacy to contribute towards academic success.

While our review suggests that a vegetarian diet may reduce the risk of depression, the existing literature on this relationship is heterogeneous. Some studies, such as those by Ocklenburg and Borawski [60], Iguacel et al. [61], and Hibbeln et al. [62], report higher levels of depression among vegetarians. Others, including Bojang and Manchana [43], and Beezhold et al. [63], report lower levels of depression. While Iguacel et al. theorize that individuals with depression may be more likely to adopt a vegetarian diet as an attempt to improve their well-being, this hypothesis requires further exploration [61]. Given the heterogeneity of the literature, additional research is needed to clarify the relationship between vegetarianism and depression.

Knowledge and Perception

Our review demonstrated heterogeneous results on the relationship between student vegetarian diet and the likelihood of counselling patients to pursue a vegetarian diet. Spencer et al. [37] and Hanna et al. [39] documented no relationship, but Morton et al. [20] and Cherpak-Castagna et al. [27] reported an association.

We theorize that there are several possible explanations for why some medical students are vegetarians but may not recommend a vegetarian diet to patients. While students may practice vegetarianism for ethical, environmental, or health reasons, they may hesitate to recommend it due to concerns about overstepping patient autonomy. Students may believe dietary changes should align with the patient’s lifestyle, socioeconomic status, culture, and readiness for change, and they may not want to be misconstrued as imposing a personal belief. Medical students may also anticipate resistance, particularly in regions where meat consumption is culturally ingrained, and may avoid suggesting vegetarianism to prevent alienating or offending patients. These theories have yet to be formally studied. 

Impact of Lifestyle

The benefits of vegetarianism may possibly result from health consciousness rather than the diet itself. Vegetarians generally smoke less and exercise more, suggesting a health-conscious lifestyle rather than inherent health benefits from the vegetarian diet [22,56,57]. Two of our included studies suggest that individuals choose a plant-based diet for health benefits, such as weight loss, indicating that motivation may influence health outcomes more than the diet itself. Suwalska et al. found that a plant-based diet is associated with greater cognitive restraint, the conscious restriction of food intake to achieve a desired body weight [29]. Similarly, Navarro-González et al. found that a higher BMI was associated with better adherence to the Mediterranean diet, acknowledging that overweight individuals may be trying to lose weight [22]. Few studies on vegetarianism control for health consciousness, and those that do, show heterogeneous results. Clarys et al. found that vegetarians still exhibited better nutrition [64], while Barr and Broughton observed no significant differences, except for deficiencies in vitamin B12 and D among vegetarians [65]. Further research should control for health consciousness to more clearly clarify the relationship between vegetarianism and cardiovascular health.

One included study by González-Sosa et al. found that adherence to the Mediterranean diet was associated with alcohol consumption at least twice per month [23]. This finding is unexpected, as other reviews largely suggest that vegetarians abstain from alcohol [66,67]. It could be argued that moderate alcohol consumption aligns with the healthy lifestyle often pursued by vegetarians. Moderate alcohol intake (i.e., one to two drinks daily) has been associated with decreased all-cause mortality and cardiovascular benefits: reduced risks of ischemic heart disease, ischemic stroke, type 2 diabetes, heart attacks, and heart failure [68,69]. It also improves lipid profiles by increasing HDL cholesterol and reducing biomarkers of thrombosis.

Conversely, moderate to heavy alcohol consumption also carries significant risks to cardiovascular health: hemorrhagic stroke, hypertension, atrial fibrillation, and cardiomyopathy [68,69]. It is also associated with alcoholic liver disease, pancreatitis, gastritis, cancer, and alcohol dependency, along with an increased risk of accidents and injuries while under the influence. Given these risks, it is surprising that medical students, who likely have heightened awareness of the health implications of alcohol, would consume it regularly while adhering to a Mediterranean diet. Further research is needed to clarify the relationship between alcohol consumption, vegetarianism, and health consciousness.

Demographics

One included study by Spencer et al. found that women are more likely than men to adopt vegetarian diets [37]. Previous literature notes that women tend to have higher disgust sensitivity, particularly toward eating animals, due to their perception of animals’ human-like intelligence [70]. Vegetarianism and food preparation are also culturally associated with femininity, leading to greater nutritional awareness among women; meanwhile, meat consumption is often linked to masculinity, with many men believing a healthy diet must include meat. Women are also more likely to consider ethical factors, such as environmental impact, animal welfare, and global food security [58]. Our review supports this explanation by including studies from Kommana et al. [34] and Spencer et al. [37]; they report that female medical students demonstrate greater nutritional awareness and prioritize this knowledge in patient counseling. These factors collectively contribute to the higher prevalence of vegetarianism among women.

Spencer et al. also found that individuals practicing Hinduism and Buddhism are more likely to follow vegetarian diets [37], a pattern rooted in the ethical and dietary principles of these religions [71-73]. The Manu Smriti, a foundational Hindu text, equates eating meat with harming living beings and likens it to murder, as it involves taking life for personal gain [74]. Hindu leaders also argue that vegetarianism is healthier and more environmentally sustainable. Similarly, Buddhism emphasizes compassion, kindness, and nonviolence, with teachings that advocate refraining from killing and treating all living creatures with respect [75]. Both religions promote love and sympathy, fostering the belief that humans share a closer affinity with animals and herbivores [74,75]. Meat consumption is often associated with Western culture, as Filippini and Srinivasan found that Hindu households exposed to more global mass media (e.g., newspapers, radio, television) are more likely to consume meat [73].

It is also worth mentioning that older students were more likely to adhere to plant-based diets [22-24]. This trend suggests that older and more knowledgeable students gravitate towards healthier dietary practices, including Mediterranean and vegetarian diets, potentially due to increased awareness and maturity.

Limitations

There were a few notable limitations in this review. There was significant variability among the included studies in methodologies, participant demographics, and definitions of plant-based diets. For instance, while some studies focused on strict vegetarian or vegan diets, others included broader dietary patterns, such as the Mediterranean diet, which we defined as vegetarian, given the discourse present in the literature. This heterogeneity complicates result synthesis and comparability.

The reliance on self-reported dietary habits and health outcomes introduces recall bias, misclassification, and social desirability bias, potentially impacting the accuracy of dietary adherence and health data. Additionally, the predominance of cross-sectional study designs limits the ability to establish causality between vegetarian diets and observed health outcomes.

Focusing on health sciences students provides valuable insights but limits generalizability. This population's unique stressors, health literacy, and dietary habits may not reflect broader demographics. Differences in institutional culture, geographic location, and vegetarian food availability likely influenced the findings. Excluding non-English studies and restricting to peer-reviewed publications may have introduced publication bias, omitting relevant research from regions where vegetarianism is more prevalent but less frequently published in English.

Moreover, the review did not thoroughly examine potential confounders, such as socioeconomic status, physical activity, or pre-existing health conditions, which may have affected outcomes. Additionally, some studies had small sample sizes, limiting statistical power and the generalizability of results. Future studies should employ standardized methodologies, objective measures of dietary adherence, and longitudinal designs while including diverse populations and accounting for confounding factors to enhance the reliability and applicability of findings.

## Conclusions

This systematic review underscores the importance of integrating comprehensive nutrition education into health sciences curricula to promote healthier lifestyles and equip future healthcare providers with the knowledge to advocate for plant-based diets. The positive physical health outcomes, such as reduced BMI and improved cardiovascular markers, highlight vegetarian diets as a cost-effective strategy for managing stress and enhancing resilience among students. Additionally, while some studies suggest that vegetarian diets may support cognitive function and mood, the relationship with depression remains inconclusive and warrants further investigation. These results highlight the potential for plant-based diets to support both physical and mental well-being, making them a valuable tool for health science students navigating high-stress environments.

Structured nutrition education plays a critical role in bridging the gap between awareness and practical application of plant-based diets. Programs that enhance students’ knowledge, confidence, and advocacy for healthier dietary habits can lead to improved adherence to beneficial diets, particularly among younger students. Addressing barriers such as cultural preferences, accessibility, and economic disparities is essential to ensure equitable adoption. These initiatives can empower future healthcare providers to integrate plant-based diets into their professional recommendations and contribute to broader public health and sustainability goals. The findings align with existing research on the cardiovascular and metabolic benefits of vegetarian diets while offering new insights into their application within a high-stress population. They emphasize the importance of exploring the nuanced impacts of vegetarian diets on academic performance and stress resilience. Future research should continue to investigate these relationships and provide evidence-based strategies for tailoring dietary recommendations to individual and community needs. By integrating these findings into educational and public health efforts, stakeholders can promote societal well-being on multiple fronts.
